# Contagion and Infection: Their Essential Nature and Characteristics

**Published:** 1883-11

**Authors:** C. T. Harris

**Affiliations:** Syracuse, N. Y.


### CONTAGION AND INFECTION: THEIR ESSENTIAL NATURE AND CHARACTERISTICS.

C. T. Harris, M. D., Syracuse, N. Y.

These command our attention to-day, and they call for an explanation of terms.

Contagion comes from " *Contingo*," or *con*, with, and *tego*, to touch, which in Latin literally means to touch with, or such diseases as come from touching or handling disease-dealing substances—the subtile matter which proceeds from a diseased person or body and which, when so handled, communicates the disease to another person. Infection and contagion are used by most medical writers as synonymous terms; others make the term contagion as any disease which comes from touch or close contact, while infection comes by imbibition, inhalation, or in any manner carried from subject to object; and hence the distinction would seem to be without exception. In the one case space intervenes between the subject and object, in the other case it does not. But to enter upon the discussion of the topic assigned, we deem it necessary first to consider the duplex nature of man, and the duplex nature of disease, and the duplex nature of the medicines necessary to meet its twofold "characteristics." Man is a bichotomous being, consisting of a body and a spirit, and if the union of the two forms a third, then would he be a trichotomous being, but as the union of the two are not lost in the third, we shall not discuss that point, as foreign to our subject of to-day.

And I refer my auditors to Genesis ii, 7: "And the Lord God formed man of the dust of the ground, and breathed into his nostrils the breath of life, and man *became* a living *soul*." When the body and spirit were united they *became* an individual, one identity—a living soul, a person. Joseph Cook says: When formed of the dust of the earth, he was a body, but not a living soul; although the members of his body were complete, symmetrical, majestic, they did not yet feel their unity with each other.

Now, if man is a duplex being and his diseases are of a duplex nature, consisting of mechanical and spiritual or vital force impaired, now, should not his remedies be also mechanical and psychological?

Man certainly is a bichotomous being, consisting of a body and spirit, which constitutes the individuality, the soul. For the body, when perfectly fitted for its tenant, had no life, no vitality, no receptivity for disease or medicine, nor has the cadaver of to-day. The breathing in the spiritual emanation from the Jehovah constituted it the *living soul*, the vital principle or *dynamization* of the creature man. The tenant took on the house as tenant until the lease expires, and the dust returns to dust as it was, and the spirit to God who gave it, to be tenant of the " house not made with hands, eternal in the heavens."

But what are mechanical or surgical diseases, and what are psychological or vital diseases?

Mechanical diseases, I understand, relate to foreign material substances in the body—wounds, poisons, etc. The first indications for the treatment of mechanical diseases, remove the cause, cleanse and adjust injured parts, and the sequence will mostly be normal by the recuperative power of nature, or vis naturæ medicatrix. But if the mechanical hiatus leaves a psychological disease it must be exterminated by established therapeutical laws.

Upon the cadaver (as we have before remarked), neither infection nor contagion nor the most virulent toxicological agent will produce vital action, for the reason that there is no spiritual tenant to be affected. The internal co-ordinating agent has departed. As well expect that plaster for house-finishing will prove reparative when placed in a decaying house as to expect that medicine or malaria will affect a diseased man without a living, co-ordinating agent. Disease acts on vital force, and to this vital force we must look for its extinction. Vitality contracts disease, and vitalized medicines must alone prove curative. Diseases of the spiritual nature of man are only amenable to vital economic laws. All the internal morbid perturbations of the invisible organism are amenable to psychological laws of spiritual existence and the abnormal symptoms are nature's *distress* flags, and wise is he who can understand and translate her distress signals. Hahnemann (*Organon*, p. 83, § 11) says: "In disease this spontaneous and immaterial vital principle pervading the physical organism is primarily deranged by the dynamic influence of a morbific agent, which is inimical to life. Only the vital principles thus disturbed can give to the organism its abnormal sensations and incline it to the irregular action which we call disease; for as an invisible principle, only cognizable through its operations in the organism, its morbid disturbances can be perceived solely by means of the expression of disease in the sensations and actions of that side of the organism exposed to the senses of the physician and bystanders; in other words, by the morbid symptoms, and can be indicated in no other manner." Sec. 10: "The material organism, deprived of its vital principle, is incapable of sensation, action, or self-preservation. It is the immaterial, vital principle only, animating the former in its healthy and morbid condition, that imparts to it all sensation and enables it to perform its functions."

Sec. 12: "It is solely the morbidly affected vital principle which brings forth disease."

The nature and characteristics of contagion and infection are to assail by their spiritual dynamics the vital functions and normal laws of life. It is a derailment of life's motor-power; but it is not an entity to be handled with Moxa, drastics, enemesis, catharsis, or emesis, by sub-cutaneous injections or corporeal bombardment, which will not cure the spirit or restore vital life; but they will destroy the subject, like Samson when he pulled Philistia's temple, with her congregated lords, about his own ears. Subject and object fell alike in the heroic downfall.

Raus' *Organon*, page 44, says: "Strictly speaking, *all* physical diseases may be traced to a violation, or at any rate to a relative weakness, of the vital force, and all diseases may, therefore, originally be considered dynamic."

Von Pommer says: "Miasmated contagion and poisons affect the blood, primarily alter and destroy the blood and with it the being."

Treviranus is of the same opinion, but thinks they act dynamically and *not* by absorption.

Steinham thinks the reaction of the blood against poison is a dynamic process.

Lobstein thinks that all diseases are in the first place dynamic.

May not, then, this difference exist between contagion and infection, contagion and mechanical disease? Hard contact may wound, and mechanical means be necessary for its expurgation, while its sequence may prove infectious; contagion may call for local treatment, as psora acorus, removed and the infection call for spiritual dynamics.

Is disease an entity? Can it be measured even by French metricles or weighed? Is infection a ponderable noxæ? Can infection or malaria be computed by masses and yet its effects puzzle the sapient pharmacologist to antidote its subtle virulence. If, then, infection is amenable to *only vital* laws, why not give our concentrated mental forces to spiritual dynamics of medicine and health? for, if you will pardon the extravaganza, we might as well strive to diet an angel with a stove-pipe as to try to remove infection or cure disease with crude doses of drugs or with drugs in crude doses.

"Oculum non curabit sine toto caput,
Nec caput sine toto corpore,
Nec totum corpus sine anima."*

"Those rules of old discovered, not devised,
Are nature still, but nature methodized."

*The eye cannot be cured without the whole head,
Nor the head without the whole body,
Nor the whole body without the spirit.

